# A New Case of Paediatric Systemic Lupus Erythematosus with Onset after SARS-CoV-2 and Epstein-Barr Infection—A Case Report and Literature Review

**DOI:** 10.3390/cimb46080509

**Published:** 2024-08-07

**Authors:** Carmen Loredana Petrea (Cliveți), Diana-Andreea Ciortea, Magdalena Miulescu, Iuliana-Laura Candussi, Sergiu Ioachim Chirila, Gabriela Isabela Verga (Răuță), Simona-Elena Bergheș, Mihai Ciprian Râșcu, Sorin Ion Berbece

**Affiliations:** 1 Faculty of Medicine and Pharmacy, University “Dunarea de Jos” of Galati, 800008 Galati, Romania; carmen.petrea@ugal.ro (C.L.P.); iuliana.candussi@ugal.ro (I.-L.C.); gabriela.verga@ugal.ro (G.I.V.);; 2 Emergency Clinical Hospital for Children “Sf Ioan”, 800487 Galati, Romania; eo111@student.ugal.ro; 3 Emergency Clinical Hospital for Children “Maria Sklodowska Curie”, 041451 Bucharest, Romania; 4 Faculty of Medicine, University Ovidius of Constanta, 900470 Constanta, Romania; sergiu.chirila@univ-ovidius.ro

**Keywords:** SARS-CoV-2, lupus, inflammation, renal, autoimmunity, paediatric patients

## Abstract

Viral infections caused by exposure to viruses such as Epstein–Barr, cytomegalovirus, or Parvovirus B19 have always been considered predisposing environmental factors for the onset of autoimmune diseases. More recently, autoimmune mechanisms such as molecular mimicry, T-cell activation, transient immunosuppression and inflammation have also been observed in cases of SARS-CoV-2 infection. Several newly diagnosed autoimmune disorders have been reported post-COVID-19, such as COVID-19-associated multisystemic inflammatory syndrome in children (MIS-C), type 1 diabetes mellitus, systemic lupus erythematosus, or rheumatoid arthritis. In this article, we present a new case of paediatric systemic lupus erythematosus (SLE) with haematological (macrophage activation syndrome), renal (stage 2), cutaneous (urticarial vasculitis) and digestive involvement, onset three and a half months post-COVID-19. In the dynamics, de novo infection generated by Epstein–Barr exposure was associated. The diagnosis was confirmed based on EULAR/ACR 2019 criteria. The aim of the article is to present a possible correlation between SARS-CoV-2 and Epstein–Barr as extrinsic factors in triggering or activating paediatric systemic lupus erythematosus. Keywords: paediatric systemic lupus erythematosus; post-COVID-19; Epstein–Barr; SARS- CoV-2; case report; paediatric patient.

## 1. Introduction

Systemic lupus erythematosus (SLE) is a potentially fatal chronic immune-mediated disease [1] that causes alterations at different stages of the immune cascade, resulting in remarkable heterogeneity of clinical manifestations, sequential, cumulative multiorgan damage over time and variable severity. The natural history of SLE ranges from an insidious, slowly progressive disease with exacerbations and remissions to an acute and rapidly fatal disease [2]. SLE is probably the autoimmune disorder with the most complex pathogenesis and clinical expression. SLE is more common in adults, and only 10–20% of cases are described in children. Childhood-onset SLE is a rare disease with an incidence of 0.3–0.9 per 100,000 children/year and a prevalence of 1.89–2.57 per 100,000 children [3]. Disease onset is described as very rare before five years of age and uncommon before adolescence, with an estimated average onset age of 11–12 years [4].

Originally diagnosed and described in the 13th century, SLE and its characteristics have been better understood due to the evolution of science. Recently, some immunological tests, such as the decrease in the level of C3 and C4 serum complements or the testing of anti-β- glycoprotein I antibodies, have become common practice [5]. The latest classification criteria for SLE, developed by the European League Against Rheumatism (EULAR)/American College of Rheumatology (ACR) in 2019, include the presence of antinuclear antibodies (ANA) as a mandatory criterion, followed by criteria grouped into seven clinical and three immunologic domains, weighted from 2 to 10 [4].

The exact aetiology of SLE is still unclear; however, it is considered a complex disease incorporating genetic, immunologic, hormonal and environmental factors [6]. The onset of SLE has also been associated with viral infections [7,8], with endogenous human retroviruses, Epstein–Barr virus, parvovirus B19, cytomegalovirus and human immunodeficiency virus type I being among the pathogens implicated in the development of the disease [9]. These agents may contribute to disease pathogenesis through different autoimmune mechanisms such as structural or functional molecular mimicry, encoding proteins that induce cross-reactive immune responses to autoantigens or modulate antigen processing, activation or apoptosis of B and T cells, macrophages, or dendritic cells [9,10]. Moreover, new research suggests a causal link between SARS-CoV-2 infection and the development of certain autoimmune diseases in genetically predisposed individuals. COVID-19 pathophysiology can lead to the presentation or exacerbation of autoimmune diseases [11] such as alopecia areata, vitiligo, systemic lupus erythematosus, vasculitis and paediatric multisystem inflammatory syndrome. Type I, II and III interferons are thought to occupy an important role in the development of autoimmune diseases post-COVID-19 by being involved in producing an appropriate response to pathogens and damaged cells in the body with impaired release of proinflammatory cytokines [12]. An increased expression of IFN-α has been observed in many patients with autoimmune pathology, especially in those with systemic lupus erythematosus [13], thus highlighting a possible association between the onset of SLE and SARS-CoV-2 infection. Although the results of studies are significant in adults, the evidence regarding the paediatric population and the causal relationship between COVID-19 and the onset of systemic lupus erythematosus in this group is very limited. 

By reviewing the scientific literature, as well as presenting a new case of paediatric systemic lupus erythematosus with onset post-SARS-CoV-2 infection, we aim to add value to the current medical knowledge regarding the correlation between COVID-19 and the onset of SLE.

## 2. Materials and Methods

We conducted a systematic review of documented cases of paediatric systemic lupus erythematosus diagnosed after the COVID-19 pandemic, covering the period from January 2021 to June 2024. All the information was searched in databases accessed through e-information platforms, such as Web of Science, PubMed, Scopus and Google Scholar. The primary inclusion criteria for the scientific literature review were as follows: studies must exclusively involve patients under 18 years of age, confirmed to have SARS-CoV-2 infection through a molecular test, and diagnosed with SLE based on EULAR/ACR classification criteria. No restrictions on the language of publication were imposed. Exclusion criteria included studies involving animals, patients over 18 years of age, or documented uninteresting results. The search algorithm used the following keywords: child, severe acute respiratory syndrome coronavirus 2, SARS-CoV-2, COVID-19, paediatric systemic lupus erythematosus, and paediatric systemic lupus erythematosus with onset post-COVID-19. 

In the presented case, we summarised and analysed the clinical manifestations, the results of paraclinical investigations, and the dynamic evolution from the onset of the first symptoms post-SARS-CoV-2 infection to the confirmation of the diagnosis of paediatric systemic lupus erythematosus. We also assessed and analysed the existence of other extrinsic factors as possible triggers of SLE and how they contributed or did not contribute to an exacerbation of the disease.

We note that this study is in accordance with the ethical standards of the institutional research committees and the Declaration of Helsinki (revised in 2013) and approved by the Medical Board of the Sf. Ioan Children’s Emergency Hospital Galati, under number C622/18.10.2023. We obtained the written informed consent of the minor patient’s guardian for the processing of medical data.

## 3. Case Description

### 3.1. Outpatient Clinical and Paraclinical Evaluation Post COVID-19

The patient, aged 14 years and 6 months, complained in May 2022 of marked fatigability, headache, mild alopecia and sudden onset amenorrhea. The patient was diagnosed with COVID-19, the Omicron variant, about three and a half months prior. SARS-CoV-2 polymerase chain reaction (RT-PCR) was positive on nasopharyngeal swabs (positive SARS-CoV-2 RNA, detection threshold 20.58), and COVID-19 was diagnosed without requiring hospitalisation. According to the World Health Organization classification criteria for COVID-19, the patient presented the asymptomatic form of COVID-19 [14,15,16]. The patient was not vaccinated against COVID-19 and had no known pathologic history.

In dynamics, the patient described new manifestations: anorexia, followed by weight loss; a first episode of macular-erythematous rash localised to the face and dorsal side of the hands; hypotrichosis associated with discolouration of eyebrows, eyelashes and hair pilosity; and then followed by the total disappearance of pilosity on the upper and lower limbs and irritability.

Paraclinical investigations revealed increases in biological markers: alanine aminotransferase (ALT: 117 U/L); aspartate aminotransferase (AST: 72 U/L); creatinine: 8.2 mg/L; total protein: 83 g/L; low values of leucocytes: 3300/mm^3^ and transferrin: 2.12 g/L; and moderate inflammatory syndrome (erythrocyte sedimentation rate-ESR: 39 mm/h; ferritin: 482 ng/mL). The non-significant ratio of serum transaminase values, as well as the serologies evaluated, ruled out a possible viral infectious cause of acute hepatitis. Also, at this initial outpatient investigation stage, an extended antinuclear antibodies (ANA) profile was performed, with anti-deoxyribonucleic double-stranded chain (DNAdc) and histones antibodies, which came back positive (Table 1). 

Endocrinologic and gynaecologic evaluation and subsequent re-evaluation, in the context of prolonged amenorrhea, did not reveal any pathological abnormalities or changes, and consequently, the diagnosis of polycystic ovarian syndrome was ruled out. Two consecutive tests with dydrogesterone were performed in order to reinstall menstruation, but with very limited success (only for one day). For the macular-erythematous eruption, antihistaminic treatment was administered for 7 days, with symptomatic improvement.

The presence of two (2) consecutive febrile outbreaks, associated with dysphagia, mouth ulcers, gingivitis with gingivorrhagia, required biological re-evaluation and serologic testing for Epstein–Barr (EBV). The results confirmed infectious mononucleosis in the seroconversion phase, with positive antibodies anti-EBV (viral capsid antigen-VCA) immunoglobulin M (IgM): 29.7 AU/mL; anti-EBV (VCA) immunoglobulin G (IgG): 696 AU/mL; and anti-EBV (EBNA): >600 AU/mL. There was mild iron deficiency anaemia (erythrocytes: 3.28 × 10^6^/µL; haemoglobin: 9.2 g/dL; haematocrit: 29%; serum iron level: 40 µg/dL; transferrin: 1.92 g/L) and elevated creatinine (9.7 mg/L). Lipid profile showed low HDL cholesterol of 30 mg/dL and LDL cholesterol of 0.73 g/L, with triglycerides and cholesterol within normal limits (Table 1). Therapeutic management focussed on symptomatic medication and restoration of iron stores, with temporary improvement of general condition and oral lesions.

From June 2022 to July 2023, the patient had a significant weight deficit of 20.4%, with a body weight reduction from 54 kg to 43 kg (11 kg). The largest weight deficits were in the intervals June–September 2022, 4 kg (7.5%), and May–June 2023, 7 kg (12.25%).

### 3.2. Clinical and Paraclinical Evaluation in Hospital-Diagnosis of SLE

In July 2023, the patient was admitted to the Sf. Ioan Children’s Emergency Clinical Hospital Galati, Romania. On admission, she had the following symptoms: fever (38.1 °C), anorexia, bilateral laterocervical and axillary micro adenopathy, slightly sensitive to palpation, dysphagia, aphtha palate, sobered tongue, dry lips, macula-erythematous rash on the anterior chest (post sun exposure in June), somnolence, pale integument and secondary amenorrhea (for about one year). The patient was extremely anxious, irritable and markedly underweight.

Following clinical examination, several differential diagnoses were considered: hematologic malignancy, neoplastic disease, autoimmune disease, inflammatory bowel disease, pulmonary tuberculosis, or acquired immunodeficiency syndrome, and consequently, extensive paraclinical evaluation and several interclinical consultations (gastroenterology, dermatologic, endocrinologic) were recommended. The dermatologic examination required differentiation with other conditions such as rosacea, lichen planus, and psoriasis [17,18,19].

Paraclinical laboratory investigations revealed severe, normochromic, normocytic anaemia (erythrocytes: 2.43 × 10^6^/µL; haemoglobin: 7.07 g/dL; haematocrit: 20.9%), leukopenia (3160/mm^3^) with lymphopenia (0.69 × 10^3^/µL), and increases in acute phase reactants (ESR: 120 mm/h and ferritin: 1047 ng/mL). Endocrinologic tests were within normal limits except for moderately elevated thyroid peroxidase antibodies (ATPO: 65.6 IU/mL). Serologies for infectious mononucleosis and COVID-19 had elevated values for the antibodies; anti-EBV (VCA) IgG: 86.6 AU/mL; anti-EBV (EBNA) IgG: 101.8 AU/mL; and anti-SARS-CoV-2 IgG: 109.4 AU/mL. The immunologic profile had pathologic values much elevated for immunoglobulin G (IgG: 3044.36 mg/dL), ANA: 1750 AU/mL, and low for serum complement C3 (40 mg/dL) and C4 (<8.0 mg/dL). There were also positive values for anti-DNA antibodies (ds > 400 IU/mL). Analysis of biochemical parameters revealed mildly elevated lactate dehydrogenase (LDH: 286 U/L) and mild hepatocytolysis (ALT: 92 U/L, AST: 124 U/L). In the context of nitrogen retention syndrome associated with the initial phase of oliguria, followed by a short transitory polyuria-polydipsia syndrome during the evolution of renal impairment, the following investigations were performed: 24 h proteinuria: 1948 mg/24 h; urea: 50 mg/dL; creatinine: 10.5 mg/L; uric acid: 7.8 mg/dL. Coagulation tests were normal except for slightly elevated D-Dimers (2.5 mg/L), molecular diagnostic and Combs’ test direct—negative (Table 1). Urine samples showed leukocyturia, Addis test without haemocytes or cylinders, and faecal samples were all negative (occult haemorrhage test, helicobacter pylori antigen, faecal calprotectin). Pharyngeal culture, nasal exudates or uroculture exams did not detect pathogenic germs.

Under applied therapeutic management, the evolution was slightly favourable, with partial return of appetite, but maintenance of a daily febrile spike (38–38.5 °C) and paraclinical was noted a mild remission of the nitrogen retention syndrome, reduction in hepatocytolysis and an increase in the posttransfusion haemoglobin level (substitution with erythrocyte components iso group, iso Rh, phenotypes).

Corroborating the paraclinical findings with the clinical elements, the EULAR/ACR 2019 classification criteria were considered met, and the patient was diagnosed with paediatric SLE. At discharge, the earliest possible referral to a paediatric nephrology specialty clinic was requested to establish the appropriate therapeutic protocol.

### 3.3. Confirmation of the Diagnosis of SLE with Lupus Nephritis—Application of Therapeutic Management

The clinical examination of the teenage patient at the nephrology clinic showed a satisfactory general status, with no cardio-respiratory or digestive pathologic changes. The patient was free of mouth ulcers and gingivitis. There were no complaints of pain (headache or arthritis). The only complaint was a macular eruption on the anterior chest (sun-exposed area). The weight on admission was 45.4 kg (a weight gain of 2.5 kg in one month) and a BMI = 17.62 kg/m^2^.

The initial report revealed normocytic, regenerative anaemia (haemoglobin: 7.2 g/dL; haematocrit 29.8%), an inflammatory syndrome (ERS: 130 mm/h; ferritin: 1440 ng/mL) and impaired renal function (Cystatin C: 1.97 mg/L; creatinine: 8.48 mg/L; urea: 8.63 mmol/L; proteinuria/creatin uria ratio: 0.25 g/mmol) (Table 1). Therefore, the renal manifestations, including the transient polyuria-polydipsia syndrome, were considered due to renal SLE manifestations. Further serologic investigations, including bacterial serologies (*Coxiella burnetii*, *Bartonella*, *Brucella*, *Leptospira*, *Rickettsia*) and viral genome sequencing (Herpesvirus type 8 [HHV8/KSHV] and Parvovirus B19), returned negative results.

Immunophenotyping was performed and revealed the following: LTCD3 + CD4 + 235 [N > 300] and NK 45 [N > 90]. Myelogram resulted in conjunction with hypergammaglobulinemia and hypoalbuminemia (serum albumin: 32 g/L), suggesting macrophage activation syndrome (MAS).

Ten days after hospitalisation, the patient developed several febrile attacks and a macula-erythematous rash on the face. There was mild thrombocytosis (550 × 10^3^/µL); increased C-reactive protein: 44 mg/dL; hypertriglyceridemia (3.3 g/L); significant decrease in glycosylated ferritin concentration (15%); polyclonal hypergammaglobulinemia; and presence of antibodies to C1q (59 U/mL) and to DNA dc (379 IU/mL) (Table 1). The decrease in glycosylated ferritin concentration below 20% raised the suspicion of STILL disease (Wissler–Fanconi syndrome), which was not confirmed in the absence of the specific clinical picture.

Dermatologic consultation, performed in the context of the rash, confirmed hypotrichosis associated with eyebrow discoloration without alopecia areata. The presence of several erythematous macules on the chest, without malarial rash, was also noted. Capillaroscopy revealed pathologic features +++ with a few pulpal red dots without necrosis and possible “alopecia areata with milder growth or protein deficiency” for the eyebrows. New blood tests for polymyositis and scleroderma antibodies came back negative.

Abdominal and trans-thoracic ultrasounds performed described “inflammatory thickening of the terminal ileum” and a “pericardial effusion lamina of approximately 7 mm”.

The clinical and paraclinical assessments supported the initial diagnosis of systemic lupus erythematosus with hematologic (macrophage activation syndrome), renal (lupus nephropathy stage 2), cutaneous (urticarial vasculitis) and digestive involvement.

A personalised therapeutic approach was decided based on the established goals in a multifactorial manner, with concomitant multisystemic and organic involvement. The autoimmune/inflammatory therapeutic plan aimed to control the macrophage activation syndrome by pulse therapy with methylprednisolone 1 g/1.73 m^2^ (850 mg) administered parenterally in bolus 3 consecutive days, followed from day 4 with prednisone 30 mg × 2/day, plaquenil 200 mg × 2/day starting from day 5, and enalapril 2.5 mg/day as a single dose.

With the reappearance of fever, rash and echo cardiac fluid lamina of ~7 mm, the prednisone dose was readjusted to 40 mg × 2/day, and 8 mg/kg of tocilizumab was introduced; both were well tolerated. Seven days after the beginning of the treatment, tocilizumab was replaced with azathioprine 20 mg/kg/day and belimumab 10 mg/kg/day, which was administered parenterally every 14 days.

Favourable clinic and biologic evolution, with remission of fever and the inflammatory syndrome and progressive improvement of lesions, led to a reduction in oral corticosteroid therapy (prednisone) to 30 mg × 2/day, associated with belimumab administered parenterally, in a single dose at 14 days (10 mg/kg/day) and azathioprine 20 mg/kg/day.

A few months after the initiation of the therapy, the polymorphous complex of manifestations was significantly reduced, appetite and menstruation returned, alopecia went into remission, recoloration of eyebrows and hairline was observed, fatigability was greatly reduced, and skin eruptive elements were absent. No new symptoms were described, and periodic clinical and paraclinical evaluations did not reveal any new pathologic features. Following corticosteroid therapy, a component of applied therapeutic management, the adolescent was about 11 kg overweight since the last hospitalisation.

## 4. Results

Through a systematic literature review, we identified a number of six relevant articles describing seven clinical cases [20,21,22,23,24,25] of the onset of paediatric SLE after SARS-CoV-2 infection. The following data were extracted from the documented reports: age, sex, method of diagnosis of COVID-19, number of days from SARS-CoV-2 infection to the onset of the first symptom attributed to SLE, clinical features, laboratory and imaging findings. 

The patients were children and adolescents aged between 9 and 13 years. Six patients were female (85.71%) [20,22,23,24,25], and only one was male (14.29%) [21]. The cases presented did not specify the vaccination status of the patients against COVID-19. The first COVID-19 vaccine was administered on 8 December 2020 to a woman in the United Kingdom, and recommendations for administration to children over 5 years of age were made by the US Centers for Disease Control and Prevention at the end of 2021 [26]. Considering the data provided, it can be estimated that in at least two of the cases [20,23], the patients were not vaccinated, as the onset of SLE occurred during the first two waves of the COVID-19 pandemic. Additionally, in another case [21], the patient presented with SARS-CoV-2 infection in the early months of 2021, prior to the recommendation to vaccinate children over 5 years of age against COVID-19. None had any significant medical history. The diagnosis of COVID-19 was confirmed by molecular testing against SARS-CoV-2. In two of the cases (28.57%), RT-PCR was positive for SARS-CoV-2 RNA [21,23], and in another two cases (28.57%), SARS-CoV-2 IgG antibodies were detected high titter [22] at presentation. One case (14.29%) relied on clinical features for diagnosis because serology was uncertain [20], while in two other cases, acute or post-COVID-19 infection was confirmed 2 months [25] and 21 days [24], respectively, before the onset of SLE. In all cases, the diagnosis of SLE was based on EULAR/ACR 2019 classification criteria. The time of onset, as well as the characteristics of clinical manifestations of SLE, were varied in the studied cases.

In one patient, the onset of SLE manifestations started in the acute phase of COVID-19 with moderate disease. In the other cases, the onset ranged from 7 or 21 days to one or two months after SARS-CoV-2 infection. In all cases, patients required hospitalisation.

Clinical manifestations characteristic of SLE were described: skin rash with atypical and/or vesiculobullous papules localised on the upper limbs or thorax, pruritic, associated with malar rash and palpebral, facial, or pedal oedema in three patients (42.85%) [20,21,22]; fever in four patients (54.17%), associated with rash in three cases (42.85%) [20,22,23]; arthralgias in one case (14.29%); and choreoathetosis in one case (14.29%) [22]. Progressive fatigue, severe pallor, and headache were reported in one case (14.29%) [24], arthralgia associated with joint swelling in two cases (28.75%) [22,24], and dysphagia, abdominal pain, and cervical adenopathy in one case (20%) [20].

Laboratory investigations revealed mild normocytic anaemia or autoimmune haemolytic anaemia in two cases (28.57%), inflammatory syndrome with elevated acute-phase reactants (CRP, VSH) in three cases (42.85%), and nitrogen retention syndrome (marked proteinuria, low serum albumin) in two cases (28.57%).

Immunologic studies revealed positive ANA and anti-DNAdc antibodies and hypocomplementemia in all cases studied (100%). Positive anti-Ro/SSA antibodies were found in two cases (28.57%), positive anti-histone antibodies in two cases (28.57%), and positive anti-RNP antibodies in one case (14.28%). No cases had positive antiphospholipid antibodies, anti-La/SSB, or anti-β-glycoprotein I antibodies, nor positive serologies for Epstein–Barr, Parvovirus B19, cytomegalovirus (CMV), HIV, viral hepatitis B and C, herpes zoster, measles, or toxoplasma. Cerebrospinal fluid analysis in one patient revealed lymphocytes, glucose of 100 mg/dL, and protein of 20 mg/dL, confirming the diagnosis of bacterial meningitis. Two days later, a bone marrow smear in the same patient showed erythroid hyperplasia with lupus erythematosus cells. Trans-thoracic ultrasonography described pleural and pericardial effusion in one patient [21], and renal biopsy confirmed lupus nephritis stage IV in two patients (28.57%). Skin biopsies in two cases described subepidermal bullae with eosinophilic infiltrate, confirming connective tissue damage [21].

Therapeutic management was based on corticosteroids (methylprednisolone, dexamethasone, or prednisone). In six cases (42.85%), hydroxychloroquine was combined with corticosteroids, and in one case, azathioprine was also used. For patients with renal involvement, cyclophosphamide (750 mg/m^2^ every 2 weeks) was introduced [21,22], and plasmapheresis was administered to patients with diffuse alveolar haemorrhage [25]. All patients achieved remission in relation to the clinical course.

## 5. Discussion

Systemic lupus erythematosus (SLE) is characterised by variability in clinical manifestation, including multiorgan involvement and a disease pattern ranging from remission to high reactivation with irreversible organ damage [27]. It is marked by the production of over 180 distinct autoantibodies, including more than 30 antiphospholipid antibodies, which are used as classification criteria due to their early presence before clinical manifestations [28,29]. 

The exact aetiology of SLE remains unclear; however, it is recognised as a complex multifactorial disease involving genetic, immunologic, hormonal, and environmental factors [30,31]. Numerous genes have been implicated in the pathogenesis of SLE, particularly components of the interferon pathways such as IRF5, STAT4, and SPP1, which likely reflect intrinsic immune deficiencies in SLE patients [32]. Viral infections such as Epstein–Barr virus (EBV), Parvovirus B19, Cytomegalovirus (CMV), and Hepatitis C virus have been associated with SLE onset, likely due to substantial immune responses [7,8].

More recently, research in the literature also suggests a link between COVID-19 and the development of certain autoimmune diseases. Since the onset of the pandemic, isolated cases of adults developing various autoimmune disorders following COVID-19 have been reported [33]. Although the underlying mechanisms remain unclear, significant increases in general inflammatory markers, such as lactate dehydrogenase (LDH), ferritin, creatinine, C3, and C4, as well as specific markers including lymphocytes, C-reactive protein (CRP), and interleukin-6 (IL-6), have been observed in COVID-19 patients [12]. The phenomenon of molecular mimicry, where foreign peptides resemble human peptides, has been proposed as a primary cause of the autoimmune responses seen in SARS-CoV-2 infections [34].

A study involving 40 adult patients with confirmed SARS-CoV-2 infection reported that 57.5% tested positive for antinuclear antibodies (ANA), 25% for antineutrophil cytoplasmic antibodies (ANCA), and anti-Saccharomyces cerevisiae antibodies (ASCA) IgA. Additionally, 17.5% had positive tests for ASCA IgG, and 12.5% had positive tests for anti-cardiolipin antibodies [33]. In a comparative study, a significantly higher incidence of autoimmune diseases was also found at six-month follow-up in the COVID-19 cohort compared to the non-COVID-19 group, with rheumatoid arthritis, systemic lupus erythematosus, inflammatory bowel disease, and type 1 diabetes mellitus being the most frequently observed conditions. Another similar study reported a 42.6% increased likelihood of developing an autoimmune disease 3–15 months after infection compared to a non-COVID-19 cohort [35].

Regarding post-COVID-19 manifestations, in February 2023, the World Health Organisation (WHO) provided the clinical case definition of post-COVID-19 (PCC) in children, according to which the condition “occurs in children and adolescents, in persons with a history of confirmed or probable SARS-CoV-2 infection, when experiencing symptoms for at least two months that initially appeared within three months of acute COVID-19. Symptoms may have a new onset after initial recovery from an acute episode of COVID-19 or may persist after the initial illness” [36]. The terms long COVID-19 and post-acute sequelae of COVID-19 (PASC), mainly used for symptoms associated with SARS-CoV-2 after four weeks, have also been associated with post-COVID-19 [37].

Based on 40 eligible studies published between 2020 and 2022, which included 12,424 people under the age of 18 years, the pooled prevalence of long-lasting COVID by specific systems and symptoms among paediatric patients was estimated to be 23.36% ([95% CI 15.27–32.53%], I^2^ = 99%; N = 17) [38]. Regarding the duration of clinical manifestations, early reports documented that between 5% and 40% of children experienced at least one persistent symptom more than 1–3 months after the initial SARS-CoV-2 infection [39,40].

Several pathogenic mechanisms have been suggested for post-COVID-19 acute sequelae, both in adults and children. The most important hypotheses were the persistence of virus and/or viral components, virus-induced tissue damage, endothelial dysfunction, coagulopathy, autonomic dysfunction, chronic inflammation, autoimmunity intestinal dysbiosis and herpetic viruses’ reactivation [41,42].

The analysis of biopsies collected from patients with COVID-19 during endoscopies revealed that 5 out of 14 patients tested positive for the SARS-CoV-2 antigen, and 3 out of 14 had positive SARS-CoV-2 RNA tests 3 months after the initial COVID-19 diagnosis [43]. Additionally, the SARS-CoV-2 nucleocapsid protein and viral RNA were found in the colon, appendix, ileum, haemorrhoids, liver, lymph nodes, and gallbladder in 5 out of 14 patients between 9 and 180 days post-COVID-19 [44]. Similarly, paediatric studies confirmed the presence of SARS-CoV-2 genome components several months after acute infection in postmortem autopsies of children with MIS-C who initially had mild or asymptomatic infections [45], including in the heart, intestine, and brain [46]. Furthermore, a multicentric study performing biopsies of intestinal lymph nodes in children with intussusception found SARS-CoV-2 particles several months after the acute infection [47].

Regarding the chronic imbalance of immune responses, studies in children showed a high variability in Treg signatures [48]. Evaluating the role of SARS-CoV-2 specific T cells in the pathophysiology of PASC, multiple adult studies revealed somewhat differing aspects. An earlier study identified a significantly lower and decreasing number of nucleocapsid-specific CD8+ cytotoxic T cells in PASC patients compared to those without PASC [49]. In contrast, another report found that patients with post-COVID-19 sequelae had reduced numbers of effector memory CD4+ and CD8+ T cells but increased expression of PD-1 (programmed cell death protein 1) on central memory cells [50]. Recent reports, however, observed significantly higher frequencies of SARS-CoV-2 specific interferon-gamma (IFN-γ) and TNF-producing T cells in PASC patients compared to those without PASC. Additionally, the presence of circulating plasma inflammatory biomarkers (IL-6 and CRP) leading to higher levels of immune activation [51], correlated with the frequency of specific T cells and PASC, concluding that PASC patients have dysregulated tissue-resident memory (TRM) CD8+ T cell responses [52].

Regarding hyperinflammation and macrophage-induced coagulopathy, studies in both adults and children suggested that these contribute to disease severity in COVID-19 patients and play a significant role in the development of sequelae and the emergence of autoimmunity [53]. Studies on PSASC patients showed a much higher frequency of intermediate CD14+ CD16+ monocytes and activated CD38+HLADR+ myeloid cells up to 8 months after mild to moderate acute COVID-19 infection compared to cohorts without confirmed SARS-CoV-2 infection [54]. 

Another aspect reported in the literature was that SARS-CoV-2 infection causes a dysregulated cytokine response with elevated expression of IFN-γ and proinflammatory cytokines such as interleukin (IL-1, IL-6, IL-7, IL-10) and tumour necrosis factor-alpha, which in turn potentially be exacerbated by the shift from Th1 to Th2 response seen in SLE [55]. 

Similar to these, the pathogenesis of SLE involves a myriad of cellular and molecular processes caused by the presence and influence of triggering factors in relation to compromised immune tolerance and immune activation through the production of hyperreactive T and B cell autoantibodies [56], the hyperproduction of circulating immune complexes, cytokines and precipitating autoantibodies on the organic cell membrane resulting in tissue inflammation, organic alteration and disease manifestation [57].

There is a growing number of reports published in the literature highlighting the development of SLE after infection with SARS-CoV-2 [2,58,59]. The presence of anti-SARS-CoV-2 antibodies and the absence of antibodies to the viruses thought to be responsible for the onset of SLE in the patients studied suggested a link between autoimmune pathologies and COVID-19. At the same time, it was noted that the onset of SLE is much delayed compared to the onset of other autoimmune diseases, with the onset occurring approximately 100 days after the diagnosis of COVID-19 [60].

Regarding the onset of SLE at an age younger than 18 years in the context of the COVID-19 pandemic, several cases have been documented in the literature, which resembles our case by similarities in clinical (rash, headache, fatigue, fever) hematologic, biochemical and immunologic manifestations.

Our patient presented a complex array of signs and symptoms progressively following SARS-CoV-2 exposure. Despite concerns about similar symptoms prior to infection, COVID-19 was confirmed by RT-PCR, and SARS-CoV-2 antibodies were detected dynamically. Notably, the patient had no prior medical history, and the sudden onset of fever, alopecia, significant weight loss, and macula-erythematous rash aligned with the clinical criteria for SLE.

The clinical manifestations of fatigue, headache, rash, and fever observed in our case are consistent with both post-COVID-19 conditions and SLE. Fatigue is a common symptom in SLE patients and a predictor of low quality-of-life scores [61,62]. Headache and rash, particularly malar rash, are also frequently reported in paediatric SLE cases [63,64,65].

In all the cases studied, including our own, SARS-CoV-2 infection was confirmed by specific molecular tests. Our patient exhibited elevated liver enzymes and inflammatory markers. Leukopenia described as the most frequent haematologic change in SLE, with 50% of patients presenting a decrease in at least one cell line [65], and it was also reported in two of the documented cases. Anaemia associated with rash, fever and fatigue was observed in two of the cases (28.57%). Proteinuria with significantly increased values was reported in two other cases (25.57%) with renal involvement, stage IV, according to renal biopsy. In terms of autoimmune screening, all the cases studied, including our own, showed elevated values of antinuclear antibodies, anti-DNA dc, and low values of serum complement C3 and C4.

In terms of therapeutic management, as in the two cases with renal involvement, the treatment was centred on corticotherapy dynamically associated with azathioprine. Additionally, the patient benefited from personalised therapy tailored to the identified pathologies and the evolutive status, starting with the initial administration of tocilizumab followed by belimumab.

A unique aspect of our case was the de novo Epstein–Barr infection approximately one year after the onset of SLE, confirmed by serologies for anti-EBV (VCA) IgG and anti-EBV (EBNA) IgG. The presence of significantly elevated anti-EBV (VCA) IgG and anti-EBV (EBNA) antibodies, along with an equivocal anti-VCA IgM antibody value, corresponds to the seroconversion of infectious mononucleosis. The significant reduction in seroprevalence for IgM anti-VCA, IgG anti-VCA and IgG anti-EBNA antibodies one month after the first EBV serologic assays comes as a supposition that the viral infection occurred 2–3 months earlier.

SLE- and EBV-induced infectious mononucleosis have been shown to have similar symptoms and clinical manifestations [66]. Epstein–Barr is a ubiquitous human virus with a productive lifecycle, mainly asymptomatic in children, and a latent phase that can last for life [67]. Serologic evidence reflecting the link between infectious mononucleosis and the development of SLE has been illustrated several times by analysing the presence of antibodies to EBNA-1, EBV-VCA and EBV-EA in sera from SLE patients. Epitopes of the latent viral protein EBNA-1 react with several lupus autoantigens, contributing to the development of lupus-specific autoimmunity through molecular mimicry [68].

Clinically, the onset of infectious mononucleosis brought an addition of new signs and symptoms, such as dysphagia, gingivitis with gingivorrhagia and laterocervical adenopathy. Together with the pre-existing complex of manifestations, these completed the clinical picture characteristic of paediatric SLE. 

One notable aspect in our case is the oscillating evolution of SLE, with periods of remission interspersed with periods of exacerbation. Secondary to the viral infection generated by EBV, anorexia worsened and led to a significant weight loss (7 kg reduction in two months), and the macula-erythematous rash with photosensitivity reappeared. Paraclinical investigations revealed moderate hematologic changes, minor inflammatory syndrome (elevated VSH and normal CRP), nitrogen retention syndrome, mild hepatocytolysis, coagulation disorders, macrophage activation syndrome, approximately two to three months after the infectious episode generated by Epstein–Barr exposure (described on admission). Anaemia, as well as an elevated value of the ESHV in parallel with a CRP within normal limits, are supportive features for the diagnosis of SLE and are included in the 11 criteria for SLE classification, with a prevalence of involvement of 50–100% [65].

With this in mind, we posed the following questions: Did Epstein–Barr contribute to the acceleration of the autoimmune process by exacerbating SLE? Did the active phase of SLE reactivate infectious mononucleosis? Or did de novo Epstein–Barr infection superimposed on a previous SARS-CoV-2 infection influence the exacerbation of the disease by hyperproduction of circulating immune complexes, cytokines, and precipitating autoantibodies?

Reports documented in the literature highlight several clinical characteristics of COVID-19 patients in contrast to those with SARS-CoV-2/EBV co-infections, such as stronger inflammatory responses and higher liver enzyme levels in patients with EBV/SARS-CoV-2 co-infection. Studies also found that patients with EBV/SARS-CoV-2 co-infection had a 3.09-fold higher risk of developing a febrile syndrome compared to patients with SARS-CoV-2 infection alone [69].

In a six-month prospective study focusing on the dynamic analysis of serologic and molecular markers of EBV infection in patients with SLE, frequent reactivation of infectious mononucleosis was observed in patients with active SLE, with the latency period occurring in most cases after six months. Transition to latent EBV infection was significantly more likely in patients with higher C3 levels. Additionally, the study provided the first evidence of the potential use of IgM anti-EA(D) antibodies in stratifying lupus patients and predicting outcomes in patients with cutaneous involvement [70].

The final aspect we wish to present is the confirmation of the diagnosis of paediatric systemic lupus erythematosus approximately 14 months after the onset of the first manifestations. The delay in referral to an immune-mediated disease such as SLE may be due to the fact that, in paediatric practice, systemic lupus erythematosus is considered the great imitator, and the absence of malarial rash at onset [71] may be a challenge for the paediatrician in its diagnosis. In our case, the variability of the symptomatology, the onset with a limited array of non-severe manifestations, the post-COVID-19 context, the absence of a malar rash at onset, and the de novo Epstein–Barr infection occurring dynamically, contributed to the delay in orientation and, consequently, the confirmation of the diagnosis of paediatric systemic lupus erythematosus. However, although the interval between the onset of manifestations and the diagnosis of SLE was approximately one year, it did not exceed the average time interval of 20 months required for the diagnosis of SLE, as reported in the literature [72,73].

There are several limitations of our study. The absence of serum C3 and C4 complement assays and ANA and dsDNA antibodies, at onset or dynamically, represented a limitation in the systematic evaluation of the patient, not only for early diagnosis but also for assessing the evolutionary phases of SLE: active or latent. As the study is retrospective, the absence of these determinations means we are unable to assess in which phase of SLE (active or latent) the infectious mononucleosis occurred. For evaluating the benefit of antiviral treatment, it would have been useful to establish, based on previously documented evidence, the existence of primary EBV infection in the personal pathological history. Dynamic assay of EBV IgM antibodies (VCA), as well as the determination of anti-EA(D) IgM antibodies or EBV viral DNA detection, are useful steps in the evaluation of the seroconversion curve. They may facilitate a more precise determination between the two possible situations, in this case, acute EBV infection that may accelerate SLE activity or EBV reactivation in the context of the acute phase of lupus.

## 6. Conclusions

This case provides a model of paediatric systemic lupus erythematosus developing post-SARS-CoV-2 infection. In the context of the COVID-19 pandemic, paediatric SLE, a long-standing immune-mediated disease, acquires new features, making it difficult to diagnose due to its multiorgan involvement and heterogeneity of clinical manifestations. Further studies are needed to assess the role of COVID-19 as a trigger of autoimmune diseases in children, with a particular focus on paediatric systemic lupus erythematosus. It is imperative to assess both the initial and dynamic evaluation of COVID-19 and SLE characteristic serologies in patients with similar situations to establish a clear causal relationship, with a view to the correct and most effective management of patients with SLE based on the potential risk they represent. 

Regarding the coexistence of SARS-CoV-2/EBV, given the complexity of the clinical picture in these situations, as well as the antigenic mimicry, followed by the production of autoantibodies that may be specific for SLE, these cases require special attention to dynamic serologic assays, representing real diagnostic challenges for the paediatrician

## Figures and Tables

**Table 1:** The pathological values of the paraclinical investigations revealed in the dynamics, in the ambulatory and during the two hospitalisations.

Biomarkers/Reference Range	Outpatient Paraclinical Evaluation	Paraclinical Evaluation Pediatric Hospital—Galati	Paraclinical Evaluation Nephrology Clinic
Erythrocytes [4.1–5.3 × 10^6^/µL]	3.28 × 10^6^	2.43 × 10^6^	
Hemoglobin [11–15 g/dL]	9.2	9.2	7.2
Hematocrit [35–45%]	29	20.9	29.6
Leukocyte [4500–13,500/mm^3^]	3300	3160	
Absolute lymphocytes [1.5–6.5 × 10^3^/µL]		0.69 × 10^3^	
No. reticulocytes [0.5–1.5%]		2.01	
Thrombocytes [150–400] × 10^3^/µL			550
Erythrocyte sedimentation rate [1–25 mm/h]	39	120	130
C reactive protein [<5.0 mg/L]	--	--	44
Ferritin [20–200 ng/mL]	482	1047	1440
Glycosylated ferritin [50–80%]	--	--	15
Serum albumin [34–50 g/L]			32
Uric acid [0–6.1 mg/dL]		7.8	
Urea [11–45 mg/dL]		50	86.3
Creatinine [5.0–8.0 mg/L]	9.7	10.5	8.48
Cystatin C [0.62–1.11 mg/L]	--	--	1.97
Dosage of urinary proteins/24 h	--	1948	
[42–225 mg/24 h]			
Creatin kinase [40–230 U/L]	--	22	76
Gama glutamine transpeptidase (GGT) [11–28 U/L]		38	
Lactate dehydrogenase [105–230 U/L]		286	274
HDL cholesterol [>50 mg/dL]	30	28	
Triglycerides [<1.50 g/L]		1.6	3.3
Total protein [57–80 g/L]	83		
Serum iron level [50–170 µg/dL]	40		
Transferrin [2.50–3.80 g/L]	1.92		
Alanine aminotransferase (ALT) [4–44 U/L]	117	92	
Aspartate aminotransferase (AST) [14–36 U/I]	72	124	
D-Dimer [0–0.55 mg/L]	--	2.52	
Anti EBV (VCA) IgG antibodies	696	86.6	--
[<17.0 AU/mL—negative >23.0 AU/mL—positive]			
Anti EBV (VCA) Ig M antibodies	29.7	<10	--
[<20.0 AU/mL—negative 20.0–40.0 AU/mL—equivocal >40.0 AU/mL—positive]			
EBV (EBNA) IgG antibodies	>600	101.8	--
[<5.0 AU/mL—negative >20.0 AU/mL—positive]			
Extended ANA profile:		--	
double stranded DNA [negative]	Positive		
Nucleosomes [negative]	Equivocal		
Histones [negative]	Positive		
Double-stranded DNA antibodies	--	>400	379
[>15 UI/mL–positive]			
Antinuclear antibodies (ANA) [0–32 AU/mL]	--	1750	--
Antibodies against C1q [<10 U/mL]	--	--	59
C1q antigens [222–354 mg/L]	--	--	85
Anti-SARS-CoV-2 IgG antibodies [>10.0 AU/mL—positive]	--	109.4	--
Anti-thyroid peroxidase antibodies (ATPO) [0–35 IU/mL]	--	65.6	
Serum complement C3 [82–160 mg/dL]	--	40	
Serum complement C4 [15–46 mg/dL]	--	<8	
IgG immunoglobulin [700–1600 mg/dL]		3044.36	Intensively
			postive 3+
25-OH-Vitamin D [30–50 ng/mL]		56.1	16
PCR EBV	--	--	Log 2.2

N.B.: Blank spaces were left for the investigations carried out that had values within normal limits, while the sign “--” was used for the investigations not carried out. Increased values are consistent with the diagnosis of systemic lupus erythematosus. Abbreviations: EBV—Epstein–Barr virus; EBNA—Epstein–Barr virus nuclear antigen; dsDNA—double-stranded deoxyribonucleic acid; HDL cholesterol—high-density lipoprotein cholesterol; RT-PCR—real-time polymerase chain reaction; SARS-CoV-2—severe acute respiratory syndrome-related coronavirus 2; VCA—viral capsid antigen.

## Data Availability

Personal medical data are publicly unavailable due to privacy or ethical restrictions, being obtained from the medical record of the patient admitted into the Emergency Clinical Hospital for Children Sf Ioan Galati.

## Abbreviations

ALT	alanine aminotransferase
ANA	antinuclear antibodies
AST	aspartate aminotransferase
COVID-19	Coronavirus Disease 2019
CRP-C	reactive protein
dsDNA	double-stranded deoxyribonucleic acid
EA(D)	diffuse early antigen
EBV	Epstein–Barr virus
EBNA	Epstein–Barr virus nuclear antigen
EULAR/ACR	European League Against Rheumatism/American College of Rheumatology
ESR	erythrocyte sedimentation rate
HDL cholesterol	high-density lipoprotein cholesterol
Ig G	immunoglobulin G
Ig M	immunoglobulin M
PASC	post-acute sequelae of COVID-19
PCC	post-COVID-19 condition
RNA	ribonucleic acid
RT-PCR	real-time polymerase chain reaction
SARS-CoV-2	severe acute respiratory syndrome-related coronavirus 2
SLE	systemic lupus erythematosus
VCA	viral capsid antigen
